# Oxaloacetate Ameliorates Chemical Liver Injury via Oxidative Stress Reduction and Enhancement of Bioenergetic Fluxes

**DOI:** 10.3390/ijms19061626

**Published:** 2018-05-31

**Authors:** Ye Kuang, Xiaoyun Han, Mu Xu, Yue Wang, Yuxiang Zhao, Qing Yang

**Affiliations:** Department of Pathogenobiology, College of Basic Medical Sciences, Jilin University, 126 Xinmin Street, Changchun 130021, China; kuangye15@mails.jlu.edu.cn (Y.K.); hanxy15@mails.jlu.edu.cn (X.H.); xumu123456@126.com (M.X.); wangy16@mails.jlu.edu.cn (Y.W.); yuxiangz17@mails.jlu.edu.cn (Y.Z.)

**Keywords:** oxaloacetate, hydrogen peroxide, carbon tetrachloride, metabolism, hepatoprotection

## Abstract

Chemical injury is partly due to free radical lipid peroxidation, which can induce oxidative stress and produce a large number of reactive oxygen species (ROS). Oxaloacetic acid is an important intermediary in the tricarboxylic acid cycle (TCA cycle) and participates in metabolism and energy production. In our study, we found that oxaloacetate (OA) effectively alleviated liver injury which was induced by hydrogen peroxide (H_2_O_2_) in vitro and carbon tetrachloride (CCl_4_) in vivo. OA scavenged ROS, prevented oxidative damage and maintained the normal structure of mitochondria. We further confirmed that OA increased adenosine triphosphate (ATP) by promoting the TCA production cycle and oxidative phosphorylation (OXPHOS). Finally, OA inhibited the mitogen-activated protein kinase (MAPK) and apoptotic pathways by suppressing tumor necrosis factor-α (TNF-α). Our findings reveal a mechanism for OA ameliorating chemical liver injury and suggest a possible implementation for preventing the chemical liver injury.

## 1. Introduction

The liver is an important metabolic organ, and its injury can be caused by viral infiltration, drugs, or toxic chemicals via infection or ingestion [1,2,3]. Chemical injury is partly due to free radical lipid peroxidation [4], which can induce oxidative stress and produce a large number of reactive oxygen species (ROS) [5,6]. ROS can induce structural and functional abnormalities in mitochondria [7]. The increasing levels of ROS can lead to dysfunction of the mitochondrial electron transport chain and tricarboxylic acid cycle (TCA cycle). The resulting lack of an energy supply causes cell injury and even death. In addition, activation of mitochondrial apoptotic pathway is also an important cause of liver injury [8,9,10].

Adenosine triphosphate (ATP) formation is mainly through two ways [11]: One is that the glucose is completely oxidized into water and carbon dioxide, and it releases a lot of ATP; the other is that the glucose is degraded to pyruvate and produces limited amount of ATP in the process with the absence of oxygen molecules [12]. Normal cells provide energy primarily through efficient aerobic metabolism in the mitochondria [13,14,15]. There is a dynamic balance between glycolysis and oxidative phosphorylation (OXPHOS). The cells will strengthen glycolysis to generate ATP if the mitochondria is damaged [16]. However, glycolysis strengthening in this situation is not enough meet the energy requirement, resulting the death in the cells. In the present study, we improved energy metabolism that was altered by hydrogen peroxide (H_2_O_2_) and carbon tetrachloride (CCl_4_) injury in the liver cells using protected mitochondria and enhanced aerobic energy supply with oxaloacetate (OA).

OA is an important intermediary in the TCA cycle and participates in metabolism and energy production. Being the key rate-limiting substrate, OA content affects the speed of the TCA cycle. Recent research has shown that adding OA to cultured neuronal SHSY5Y cells can enhance both ATP production and respiratory fluxes [17]. Another related study reported that OA promotes brain mitochondrial biogenesis and activates the insulin signaling pathway. Thus, OA contributes to bioenergetic fluxes and biosynthesis [18].

In our study, we used H_2_O_2_ or CCl_4_ as a model of liver injury in vivo and in vitro. We found that OA had good hepatoprotective effects by preventing chemical damage to the liver. This protection mechanism maintains the structural and functional integrity of the mitochondria by reducing ROS production and enhancing energy metabolism. In addition, OA further enhanced hepatoprotection by inhibiting the mitogen-activated protein kinase (MAPK) pathway and mitochondrial apoptosis.

## 2. Results

### 2.1. Protective Effect of Oxaloacetate (OA) on the Human Normal Liver Cells (LO-2 Cells) against Hydrogen Peroxide (H_2_O_2_) Injury

**Figure 1:**
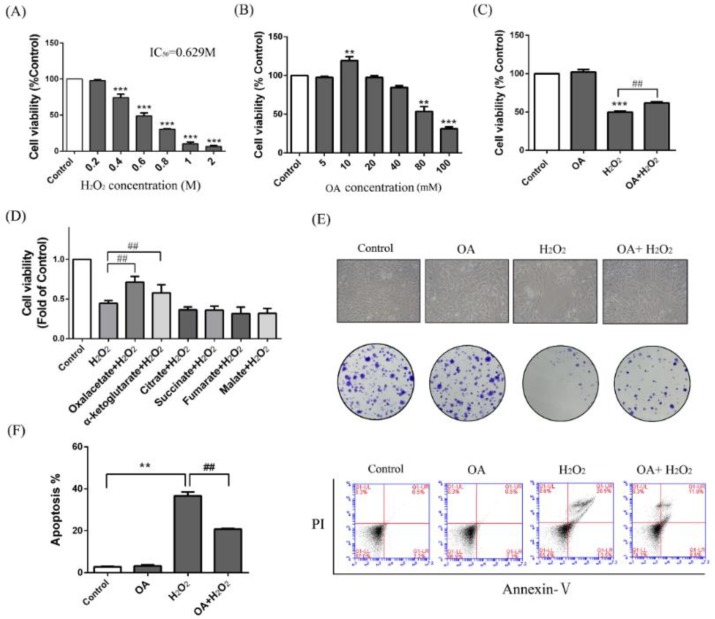
Hepatoprotective effect of oxaloacetate (OA) on human normal liver cells (LO-2 cells) against hydrogen peroxide (H_2_O_2_) injury. (**A**) The LO-2 cells were treated with 0.2, 0.4, 0.6, 0.8, 1, and 2 M H_2_O_2_ for 24 h. Cell viability was measured using an 3-(4,5)-dimethylthiahiazo (-z-y1)-3,5-di- phenytetrazoliumromide (MTT) assay; (**B**) MTT assay. The LO-2 cells were treated with 5, 10, 20, 40, 80, and 100 mM OA for 24 h; (**C**) MTT assay. Groups were as described in “Methods: Cell line and Reagents”; (**D**) MTT assay. The LO-2 cells were pretreated with 10 mM different TCA substrates before treatment with 600 mM H_2_O_2_; (**E**) Cell microscopic morphology (200×) and colony formation. Groups were as described in “Methods: Cell line and Reagents”; (**F**) Annexin V-PI assay. Groups were as described in “Methods: Cell line and Reagents”. The column chart shows the percentage of apoptotic cells in each group. The experiments were repeated at least three times. The results were presented as the mean ± standard deviationc (SD). ** *p* < 0.01, *** *p* < 0.001 compared with the control group and ^##^ *p* < 0.001 compared with H_2_O_2_-treated group.

As indicated in Figure 1a, the human normal liver cells (LO-2) cells were treated with different concentrations of H_2_O_2_ for 24 h, and the 50% inhibitory concentration was found to be 629 mM (Figure 1a). Therefore, we used the concentration of 600 mM as a model of H_2_O_2_ liver injury. The activity of the LO-2 cells was increased 15.23 ± 8.14% of the control group with 10 mM OA treatment (Figure 1b). As indicated in Figure 1c, with OA pretreatment, the cell activity was increased to 1.23 ± 0.35-fold of the H_2_O_2_ group. When we further compared OA with other TCA substrates, only OA and α-oxoglutarate had a protective effect, and the protective effect of OA was more obvious than that of α-oxoglutarate (Figure 1d). With OA pretreatment, the cellular state was better than that of the H_2_O_2_ group under the optical microscope (Figure 1e). We further examined the apoptotic level using Annexin V/PI assay. The result of flow cytometry (FCM) showed that the number of apoptotic cells in the protection group (OA + H_2_O_2_) was lower than those in the H_2_O_2_ group (56.54 ± 6.31% of the H_2_O_2_ group) (Figure 1f). Meanwhile, colony formation assay indicated that the LO-2 cells in the H_2_O_2_ group were unable to effectively form clones. After pretreatment with OA, the clonal number was increased (Figure 1e). Moreover, the addition of OA alone caused no injury to the LO-2 cells. These results indicate that OA had protective effects on H_2_O_2_-induced injury on the liver cells.

### 2.2. Effects of OA on Liver Weight and Liver Index

**Figure 2:**
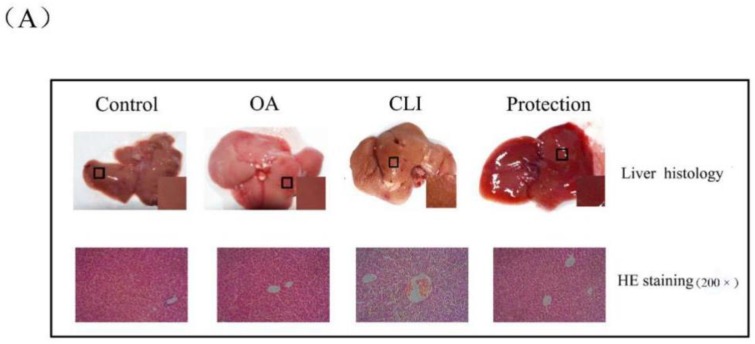

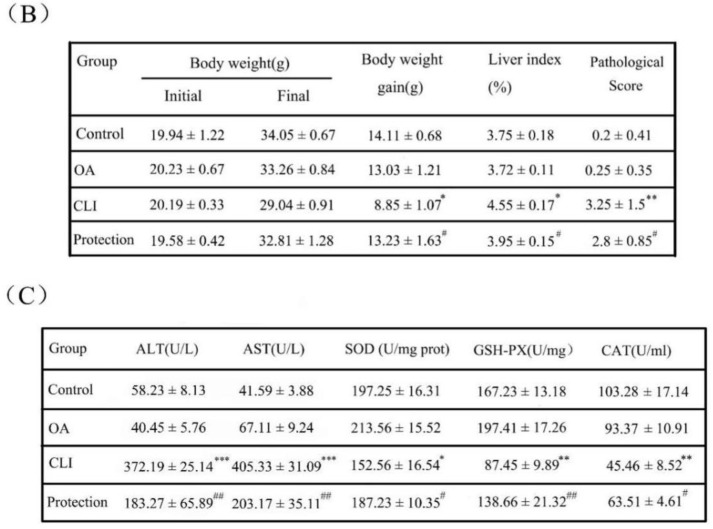
Hepatoprotective effect on carbon tetrachloride (CCl_4_)-induced chronic liver injury in mice. (**A**) Liver tissue of mice and corresponding hematoxylin-eosin (HE) staining. Groups were as described in “Methods: animal”. The tissue was enlarged locally and diffusion miliary dots were observed in the chronic liver injury (CLI) group; the HE staining was 200×; (**B**) Body weight gain, liver index, and pathological score. Groups were as described in “Methods: animal”; (**C**) The activity of alanine transaminase (ALT), aspartate aminotransferase (AST), superoxide dismutase (SOD), glutathione peroxidase (GSH-PX), and catalase (CAT). Groups were as described in “Methods: animal”. Data was the mean ± SD of *n* = 10 mice/group. * *p* < 0.05, ** *p* < 0.01, *** *p* < 0.001 compared with the control group and ^#^ *p* < 0.05, ^##^ *p* < 0.01 compared with the CLI group.

The mice of control group received intraperitoneal injections of olive oil (10 mL/kg body weight) for four weeks (twice a week). The mice of chronic liver injury (CLI) group received intraperitoneal injections of 0.1% CCl_4_ solution in olive oil for four weeks (twice a week). The OA group received intraperitoneal injections of 10 mM OA solution in olive oil for four weeks (twice a week). The mice of protection group received intraperitoneal injections of 10 mM OA solution for 1 h prior to injections of 0.1% CCl_4_ solution in olive oil for four weeks (twice a week). As indicated in Figure 2b, the increase in weight of the mice in the CLI group (8.85 ± 1.07 g) was 68.12 ± 0.13% of the mice in the control group (14.11 ± 0.68 g). The increase in weight of the mice in the protection group (13.23 ± 1.62 g) compared with the control group was not obviously changed. The liver index was the ratio of liver weight to body weight. In the CLI group, the mice livers showed that the liver index score was increased 1.07 ± 0.08-fold of the control group. In the protection group, the liver index was decreased 86.97 ± 0.06% of the CLI group. The pathological score showed that the mice livers in the CLI group appeared obvious pathological lesion, which was alleviated in the protection group. These results suggest that OA can alleviate the CCl_4_-induced chronic liver injury in vivo. 

### 2.3. Hepatoprotective Effect of OA in Liver Histopathology

As shown in Figure 2a, the liver morphology of the control group and the OA group was normal: The structure was intact, the capsule was smooth, the color was ruddy, and the texture was soft. However, the liver injury of the CLI group was obvious: The liver was yellowish-brown and swollen, the texture was fragile, the edge was blunt, the capsule was tense, and the miliary granules were diffused. Compared with the control group, the degree of lesion in the protective group was relieved. We further performed hematoxylin-eosin (HE) staining on tissue sections. In the control group and OA group, the hepatic lobule structure was clear, the cell morphology was normal, and the hepatic cords were arranged regularly. In the CLI group, the liver exhibited a high degree of ballooning degeneration and accumulation of fatty vacuoles and inflammatory cells. In the protective group, the severity of the lesions was reduced. The liver tissues of all groups were assigned a pathological score according to the Inspection and Assessment Standard for Health Food issued by the Ministry of Health of the People’s Republic of China. There were almost no lesions in the control group and the OA group. The average pathological score of the CLI group reached 3.25. Compared with the CLI group, the average pathological score of protection group reached 2.8 (Figure 2b). Based on the histology and pathology results, OA had protective effects on chronic liver injury caused by CCl_4_.

### 2.4. Effects of OA on Liver Enzymes

Activity of alanine transaminase (ALT) and aspartate aminotransferase (AST) is important indicator of liver injury. As shown in Figure 2c, the activity of ALT and AST in the CLI group was increased to 6.44 ± 0.38 and 9.76 ± 0.13-fold of the control group and was alleviated with OA pretreatment in the protection group (2.89 ± 0.83 and 4.86 ± 0.32-fold of the control group). Superoxide dismutase (SOD), glutathione peroxidase (GSH-PX), and catalase (CAT) are involved in ROS scavenging and peroxides breakdown [19,20]. We therefore tested the activity of SOD, GSH-PX, and CAT in the tissues. As shown in Figure 2c, the activity of SOD, GSH-PX, and CAT in the CLI group was clearly decreased to 77.32 ± 0.85%, 52.2 ± 0.6%, and 40.91 ± 0.81% of the control group. With OA pretreatment, the activity of SOD, GSH-PX, and CAT was increased to 1.22 ± 0.17, 1.58 ± 0.24, and 1.46 ± 0.1-fold of the CLI group (Figure 2c). Thus, we suggest that OA contributed to the recovery of the activity of the liver enzymes to the normal levels.

### 2.5. Protective Effect of OA on Mitochondria

**Figure 3:**
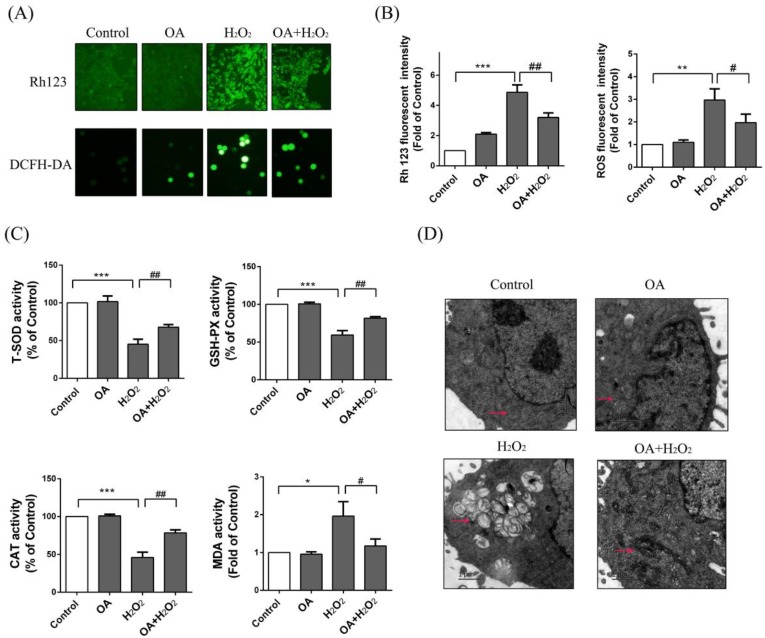
Effects of OA on reactive oxygen species (ROS) and morphology of mitochondria. (**A**) The membrane potential changes and ROS production were detected by Rh123 and DCFH-DA probes respectively by fluorescence microscope (200×). Groups were as described in “Methods: Cell line and Reagents”; (**B**) The fluorescence intensity of Rh123 and ROS was measured by FCM; (**C**) The activity of SOD, GSH-PX, CAT, MDA. Groups were as described in “Methods: Cell line and Reagents”; (**D**) The morphology of mitochondria by transmission electron microscopy. Groups were as described in “Methods: Cell line and Reagents”. Pink arrows point to mitochondria. The experiments were repeated at least three times. The results were presented as the mean ± SD. * *p* < 0.05, ** *p* < 0.01, *** *p* < 0.001 compared with the control group and ^#^ *p* < 0.05 and ^##^ *p* < 0.01 compared with the H_2_O_2_ group.

Rh123 is an indicator of mitochondrial transmembrane potential [21,22]. Mitochondrial injury cause changes in membrane potential. The FCM results showed that the fluorescence intensity of Rh123 in the H_2_O_2_ group was increased to 4.86 ± 0.44-fold of the control group, and normalized a bit with OA pretreatment (OA + H_2_O_2_ group) (3.24 ± 0.36-fold of the control group) (Figure 3b). ROS accumulation in the H_2_O_2_ group was increased 2.96 ± 0.36-fold of the control group and alleviated with OA pretreatment (OA + H_2_O_2_) (2.06 ± 0.64-fold of control group) (Figure 3b). We further studied the effects of OA on antioxidant enzymes. As shown in Figure 3c, the activity of SOD, CAT, and GSH-PX in the H_2_O_2_ group were clearly decreased to 45.32 ± 5.1%, 45.62 ± 6.43%, and 59.34 ± 6.21% of the control group. With OA pretreatment, the activity of SOD, GSH-PX, and CAT were increased to 1.55 ± 0.51, 1.48 ± 0.35, 1.47 ± 0.81%-fold of with the H_2_O_2_ group. We further examined the content of MDA in the LO-2 cells. The results showed that the amount of MDA in the H_2_O_2_ group was increased to 1.95 ± 0.43-fold of the control group and was decreased to 60.32 ± 15.35% of the H_2_O_2_ group with OA pretreatment. We then observed the morphology of mitochondria using transmission electron microscopy. As shown in Figure 3d, following H_2_O_2_ treatment, most of the LO-2 cells had swollen, the mitochondria underwent vacuolization, and the cristae was absent or broken. After OA pretreatment, the mitochondrial vacuolization was almost invisible, the number of mitochondria was increased, the mitochondria were pyknotic, and the matrix color was deepened. These results show that OA was able to reduce the oxidative injury caused by H_2_O_2_ and maintain the integrity of the mitochondrial structure.

### 2.6. Effect of OA on Glycolysis

**Figure 4:**
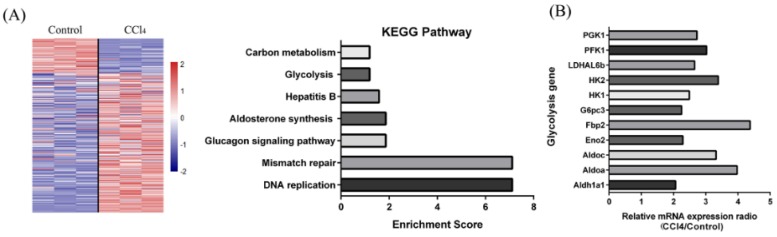

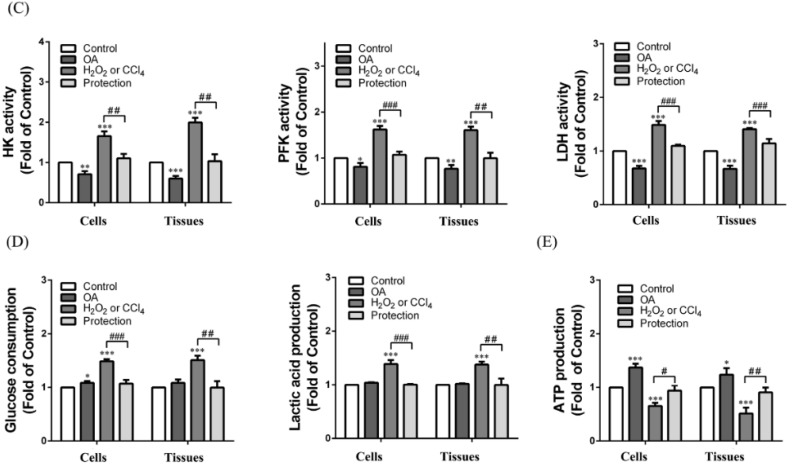
Effect of OA on glycolysis. (**A**) The enrolled genes were conducted the unsupervised hierarchical clustering with edgeR Package (left panel). Partial kyoto encyclopedia of genes and genomes (KEGG) pathway enrichment (right panel); (**B**) The column diagram represents mRNA expression levels of different glycolytic genes (CCl_4_ vs. Control); (**C**) The enzymatic activity of hexokinase (HK), phosphofructokinase (PFK), and lactate dehydrogenase (LDH) in the cells and tissues. Groups were as described in “Methods: Cell line and Reagents, animals”; (**D**) Glucose consumption and lactic acid production in the LO-2 cells and tissues. Group were as described in “Methods: Cell line and Reagents, animals”; (**E**) ATP production in the LO-2 cells and tissues. Groups were as described in “Methods: Cell line and Reagents, animals”. The experiments were repeated at least three times. The results are presented as the mean ± SD. * *p* < 0.01, ** *p* < 0.01, *** *p* < 0.001 compared with the control. ^#^ *p* < 0.05, ^##^ *p* < 0.01, ^###^ *p* < 0.001 compared with the H_2_O_2_ or CCl_4_ group.

Using KEGG pathway enrichment, we found that the expression of glycolysis-related enzymes was markedly elevated with CCl_4_ treatment in the liver (Figure 4a,b). We hypothesized that when the mitochondria were injured, it must have increased glycolysis to provide energy and adjust for any substance deficiencies. With H_2_O_2_ or CCl_4_ treatment, the enzymatic activity of hexokinase (HK), phosphofructokinase (PFK), and lactate dehydrogenase (LDH) were increased to 1.84 ± 0.01, 1.5 ± 0.08, and 1.41 ± 0.32-fold of the control group in the cells and 1.93 ± 0.06, 1.86 ± 0.12, and 1.48 ± 0.41-fold of the control group in the tissues (Figure 4c). The increase of enzyme activity resulted in the increase of glucose consumption and lactic acid production in the cells and tissues (Figure 4c). With OA pretreatment (protection group), the enzyme activity of HK, PFK, and LDH returned to normal in the cells and tissues. With OA pretreatment, glucose consumption and lactic acid production were decreased to 79.27 ± 7.28% and 85.71 ± 4.28% of the H_2_O_2_ group in the cells, and were decreased to 64.21 ± 3.52% and 53.22 ± 5.51% of the CCl_4_ group in the tissues. (Figure 4c). However, glycolysis in this situation was not enough meet the energy requirement. We further examined the generation of ATP in the cells and tissues. The results showed that ATP output was increased to 1.94 ± 0.33 and 2.41 ± 0.28-fold of the control group with OA treatment in the cells and tissues. ATP output was increased to 1.24 ± 0.25-fold of the H_2_O_2_ group and 1.65 ± 0.48-fold of the CCl_4_ group with OA pretreatment in the cells and tissues (Figure 4e). Here, we suggest that OA led to an increase in ATP production and a decrease in anaerobic energy supply through restoration of aerobic energy supply.

### 2.7. Effect of OA on Tricarboxylic Acid (TCA) Cycle and Electron Transport Chain

**Figure 5:**
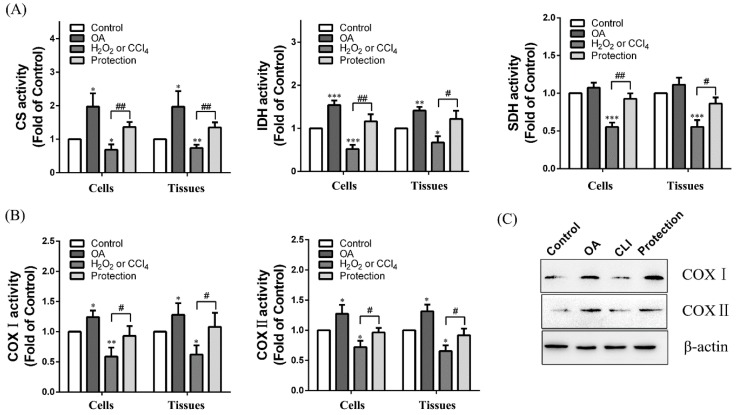
Effects of OA on TCA cycle and electron transport chain. (**A**) The enzymatic activity of citrate synthase (CS), isocitrate dehydrogenase (IDH), and succinate dehydrogenase (SDH) in the cells and tissues. Groups were as described in “Methods: Cell line and Reagents, animals”; (**B**) The activity of complex (COX) I and II in the cells and tissues. Groups were as described in “Methods: Cell line and Reagents, animals”; (**C**) Western blot assay. Groups were as described in “Methods: animals”. The experiments were repeated at least three times. The results are presented as the mean ± SD. * *p* < 0.05, ** *p* < 0.01 and *** *p* < 0.001 compared with the control. ^#^ *p* < 0.05, ^##^ *p* < 0.01 compared with the H_2_O_2_ or CCl_4_ group.

We explored the impact of OA on the TCA cycle in the cells and tissues. With OA treatment, activity of citrate synthase (CS), isocitrate dehydrogenase (IDH) and succinate dehydrogenase (SDH) were increased 1.94 ± 0.41, 1.72 ± 0.18, and 1.08 ± 0.09-fold of the control group in the cells and 1.92 ± 0.52, 1.51 ± 0.14, and 1.11 ± 0.27-fold of the control group in the tissues. Meanwhile, OA was able to relieve the decrease of enzyme activity which was caused by H_2_O_2_ or CCl_4_ (Figure 5a). We further explored the impact of OA on the electron transport chain. With OA treatment, the activity of COX I and II were increased to 1.31 ± 0.05 and 1.23 ± 0.13-fold of the control group in the cells, and 1.32 ± 0.19 and 1.3 ± 0.15-fold of the control group in the tissues. With OA pretreatment (protection group), the activity of COX I and II was increased to 1.61 ± 0.41 and 1.29 ± 0.43-fold of the H_2_O_2_ group in the cells and 1.71 ± 0.37, and 1.33 ± 0.39-fold of the CCl_4_ group in the tissues (Figure 5b). The results of Western blot were consistent with the activity test (Figure 5c). These results indicate that OA was able to promote the TCA cycle and electron transport chain to supplement the ATP deficiency resulting from chemical injury.

### 2.8. Effect of OA on Mitogen-Activated Protein Kinase (MAPK) Pathway

**Figure 6:**
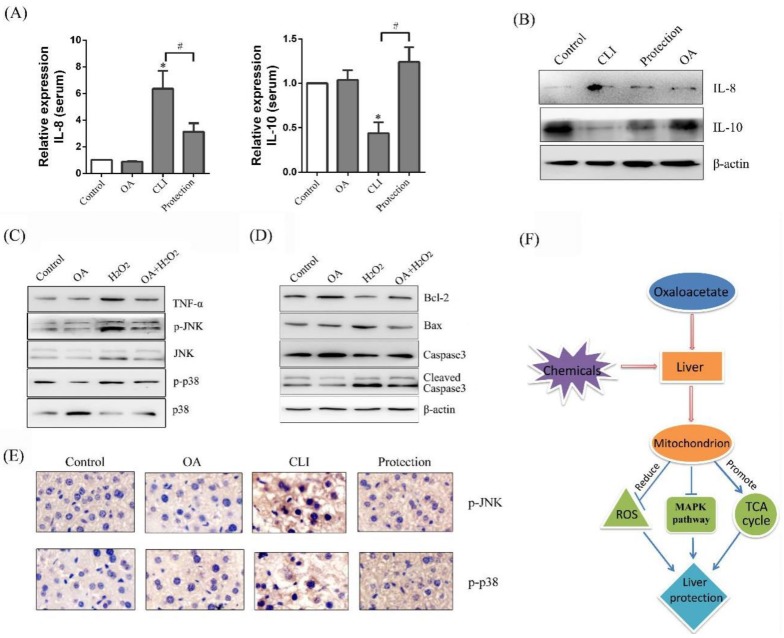
Effects of OA on mitogen-activated protein kinase (MAPK) pathway. (**A**) ELISA of IL-8 and IL-10 in the serum. Groups were as described in “Methods: animals”. The experiments were repeated at least three times. The results are presented as the mean ± SD. * *p* < 0.05, compared with the control. ^#^ *p* < 0.05, compared with the CLI group; (**B**) Western blot of interleukin-8 (IL-8) and IL-10 in the serum. Groups were as described in “Methods: animals”; (**C**) Western blot of tumor necrosis factor-α (TNF-α), p-JNK, N-terminal kinase (JNK), p-p38, and p38 mitogen-activated protein kinase (p-38) in the LO-2 cells. Groups were as described in “Methods: Cell line and Reagents”; (**D**) Western blot of Bcl-2, Bax, Caspase3, Cleaved-caspase3, β-actin in the LO-2 cells; (**E**) Immunohistochemistry of p-JNK, p-p38 in the tissues (400×). Groups were as described in “Methods: animals”; (**F**) Schematic presentation of hepatoprotective effect of OA in the liver.

Chemokines participate in the recruitment and mediation of inflammatory cells through binding with chemokine receptors [23]. IL-8 is a chemotaxis cytokine that promotes chemotaxis on inflammatory cells. High IL-8 levels are tested in the injured liver. In our study, with CCl_4_ treatment (CLI group), the content of IL-8 was increased to 6.35 ± 1.34-fold of the control group in the serum. With OA pretreatment (protection group), the content of IL-8 was decreased to 51.8 ± 19.37% of the CLI group (Figure 6a). We next detected the protein expression of IL-8 in the tissues by Western blot assay. The same results were showed by Western blot as enzyme-linked immuno sorbent assay (ELISA) (Figure 6b). Cytokines, a class of secretory proteins, produced by lymphocytes, mononuclear macrophages, and fibroblasts, promotes the growth and differentiation of other cells, enhancing the immune response and metabolism of inflammatory cells [24]. IL-10 is an important immunomodulatory cytokine. Its biological function is mainly to limit inflammatory response and regulate the differentiation and proliferation of immune cells. In our study, with CCl_4_ treatment (CLI group), the content of IL-10 was decreased to 43.67 ± 12.22% of the control group in the serum. With OA pretreatment (protection group), the content of IL-10 was increased to 3.02 ± 1.11-fold of the CLI group (Figure 6a). We next detected the protein expression of IL-10 in the tissues by Western blot assay. The same results were showed by Western blot as ELISA (Figure 6b). The elevated ROS and inflammatory factors were induced by H_2_O_2_ or CCl_4_, which activated the MAPK signal pathway. The mammalian MAPK family consists of extracellular regulated protein kinases(ERK), N-terminal kinase (JNK), and p38 mitogen-activated protein kinase (p38 MAPK). As shown in Figure 6a, the expression of tumor necrosis factor-α (TNF-α) was increased, and JNK and p38 were activated in the H_2_O_2_ group. OA was able to reduce the expression of TNF-α and inhibit H_2_O_2_-induced JNK and p38 phosphorylation (p-p38) (Figure 6c). In addition, as the MAPK pathway was activated, the corresponding Bax expression was increased, Bcl-2 expression was decreased and caspase3 was activated in the H_2_O_2_ group. However, OA pretreatment inhibited the MAPK pathway and then suppressed expression and activation of apoptotic-related pathways (Figure 6c,d). The results of p-JNK and p-p38 immunohistochemistry in the tissues were the same as that of the Western blot (Figure 6e).

## 3. Discussion

Energy output in the cells is a response of the cell to the energy requirement. Thus, ATP production of the cells varies with environment changes of the cells [25]. ATP in the cells is produced mainly by the glycolysis and OXPHOS [26,27]. The proportion of the two varies with the environment in which the cells live. In normal aerobic cells, ATP is produced mainly by the OXPHOS providing 70% of the energy required for cellular metabolism. The glycolysis occurs under hypoxic situations. The glycolysis and OXPHOS interact with each other to maintain cellular energy balance [28,29]. Assuming that the total ATP is a constant, the glycolysis would increase in order to maintain cellular energy balance if the OXPHOS function is impaired, and the glycolysis would be maintained at a lower level if the OXPHOS is functioning properly [30]. Therefore, when the cells are in a harmful environment, the homeostasis of cell energy metabolism can be maintained by modulating the level of the glycolysis and OXPHOS.

Liver injury induced by chemical reagents is mainly related to excessive formation of free radicals and peroxidation [31,32]. Lipid peroxidation can cause damage to the structure and function of mitochondria, accompanied by disorders of energy metabolism, and substance metabolism [33,34]. For our study, as the endogenous compound, OA had efficient protective effects on H_2_O_2_ or CCl_4_ induced liver injury in vivo and in vitro. On one hand, OA was able to remove excess ROS and protect the integrity of the mitochondrial structure. On the other hand, the hepatic cell was needed to accelerate energy metabolism to supplement the energy deficiency after liver cells were injured. A small dose of OA could enhance energy metabolism by promoting TCA circulation and accelerating OXPHOS. However, the limitation in using gene microarrays to study cellular metabolism should be taken into account, as the expression of mRNAs and proteins are not parallel [35]. The metabolomics is a better way to study cell metabolic changes. 

In addition, OA promoted the proliferation of LO-2 cells. It is well understood that oxaloacetic acid is transported into the mitochondrial matrix via the malate shuttle system in the form of malate or aspartate. It was interesting that both malate and aspartate had no effect on the proliferation of hepatocytes [36]. In our experiments, the protein phosphorylation level of JNK and p38 pathway was decreased with OA treatment compared with the control group. It is likely that the extra-mitochondrial oxaloacetic acid was important signaling molecule for growth induction.

Our previous research has reported that 75 mM OA caused a death of HepG2–human hepatic carcinoma cells, while 20–30 mM OA increased HepG2 cell proliferation [37]. Similar results were also observed on the normal liver LO-2 cells in the present experiment. As shown in Figure 1b, the viability of LO-2 cells was increased when the dose of OA was at 10 mM and began to decrease when the dose of OA was increased to 40 mM. Thus, the hepatoprotective effect of OA can be achieved only at lower dose of OA.

In a recent preclinical study, OA in capsule form was administered to patients for the treatment of Alzheimer’s disease. The capsules contained 100 mg OA and 150 mg of ascorbic acid. In addition to OA’s proposed use in the treatment of AD and diabetes, recent preclinical research has also tested OA which may be used for therapy of traumatic brain injury, stroke, and amyotrophic lateral sclerosis [38,39,40,41]. Chemical liver injury is a common liver disease and usually caused by the ROS generated by toxic chemicals. The present study demonstrated that OA ameliorated chemical liver injury via oxidative stress reduction and enhancement of bioenergetic fluxes and implied that OA was a potential therapeutic reagent for the treatment of chemical-induced liver injury. However, some limitations for clinical use of OA were acknowledged. It was reported that 100 mg of OA failed to increase the plasma concentration of OA and large doses of OA could lead to hypoglycemia [42,43]. Because solutions of OA are unstable, it could be easily decomposed into other products in the plasma. To enhance the stability of OA in the plasma, we may need to optimize the type and dissolution conditions of the solvent for OA or modify the molecular structure of OA. OA is an endogenous metabolite, and could cause metabolic disorder if OA is over dosed. One of the options to solve this problem may be to use metabonomics to detect the effects of OA on all metabolic products in the plasma, so as to determine a safe and effective dosage range of OA. Although the clinical application of OA is still in its infancy, we believe that OA has a wide variety of prospective clinical applications in liver protection.

## 4. Materials and Methods

### 4.1. Cell Line and Reagents

The normal hepatocyte LO-2 cell line was purchased from the cell bank of Shanghai Institute of Biochemistry and Cell Biology. Oxaloacetic acid was purchased from Sigma (Ronkonkoma, NY, USA). OA solution was prepared by dissolving oxaloacetic acid in phosphate-buffered saline (PBS) and the pH was adjusted to approximately 7.0 with NaOH. Because OA was relatively unstable in solution, the solution was prepared immediately before each use. H_2_O_2_ and CCl_4_ were purchased from Sigma (USA).

LO-2 cells were treated PBS for 24 h as the control group. LO-2 cells were treated with 10 mM OA for 24 h as the OA group. LO-2 cells were treated with 600 mM H_2_O_2_ for 24 h as the H_2_O_2_ group. The LO-2 cells were treated with 10 mM OA for 1 h prior to treat with 600 mM H_2_O_2_ for 24 h as the protection group (OA + H_2_O_2_). 

### 4.2. Animals

Forty male mice aged 4–5 weeks old and weighing 18–20 g were purchased from the Experimental Animal Center at Jilin University (China). The mice were housed in plastic cages (four mice per cage) at 24 ± 2 °C and 60% humidity with a 12 h–12 h light–dark cycle and had ad libitum access to water and standard mouse chow. The study protocol was approved by the Ethics committee of the Jilin University Health Science Center (Code 22, 15 March 2017). During the experiment, animal handling and care were carried out according to international laboratory animal use and care guidelines.

After one week on the basal diet, the mice were fasted for 24 h before the experiment. The control group received intraperitoneal injections of olive oil (10 mL/kg body weight) for four weeks (twice a week). The chronic liver injury (CLI) group received intraperitoneal injections of 0.1% CCl_4_ solution in olive oil for four weeks (twice a week). The OA group received intraperitoneal injections of 10 mM OA solution in olive oil for four weeks (twice a week). The protection group received intraperitoneal injections of 10 mM OA solution for 1 h prior to injections of 0.1% CCl_4_ solution in olive oil for four weeks (twice a week). The mice in each group were weighed daily.

### 4.3. Cell Viability and Colony Formation

Cell viability was assessed using MTT method. Briefly, the cells were grown in 100 μL dulbecco’s modified eagle medium (DMEM) at a density of 8 × 10^4^ cells per well in a 96-well plate. After 24 h, the cells were adherent with free serum for 12 h. After the cells had been processed, 0.01 mL the 3-(4,5-dimethyl-2-thiazolyl)-2,5-diphenyl-2-*H*-tetrazolium bromide (MTT) solution (5 mg/mL) was added to each well and incubated at 37 °C for 4 h. Formazan crystals were then dissolved with dimethyl sulfoxide (DMSO), and the optical densities at 490 nm were measured using a microplate reader (Bio-Tek, Winooski, VT, USA). Three hundred LO-2 cells per well were seeded in 6-well plates. After 7–10 days, colonies were fixed in methanol and stained with 0.1% crystal violet, and the number of colonies was counted.

### 4.4. Analysis of Cell Apoptosis

Apoptosis of the LO-2 cells was analyzed using the Annexin V: PI Apoptosis Detection Kit I (BD Biosciences, San Jose, CA, USA). Briefly, the cells were resuspended in 100 μL of binding buffer with 5 μL Annexin V and 10 μL propidium iodide (PI), and then incubated in the dark at room temperature for 15 min. The samples were analyzed by flow cytometry (FCM) (Beckman Coulter, Miami, FL, USA).

### 4.5. Histology and Immunohistochemistry

Histological identification was performed following a midline laparotomy to remove the liver after euthanizing the mice. Liver tissues were morphologically identified and weighed. The tissue samples were fixed in 10% formalin for 24 h, dehydrated, and embedded in paraffin. Hematoxylin-eosin (HE) staining was performed on 4 μm thick sections of the livers. The samples were deparaffinized in xylene and rehydrated using a series of graded alcohol solutions. The slides were blocked with 10% goat serum before antibody incubation. The samples were incubated overnight with a primary antibody followed by incubation with a secondary antibody and analysis by optical microscopy (Nikon, Minato-ku, Japan, TS100-F).

### 4.6. Western Blot

The cells were washed twice with cold PBS after collection, then lysed in Radio Immunoprecipitation Assay (RIPA) buffer (Sigma, USA). The reaction was performed on ice for 30 min, and then the lysate was centrifuged at 4 °C at 12,000 rpm for 30 min. The supernatant was saved, and the protein concentration of the samples was measured using the bicinchoninic acid (BCA) method. The proteins were resolved on a 10% SDS-polyacrylamide gel electrophoresis (SDS-PAGE) gel and transferred to a polyvinylidene difluoride (PVDF) membrane. The membrane was blocked overnight with 5% non-fat milk, and the primary antibody was added to incubate for 2 h at room temperature. After incubation with primary antibody and the secondary antibody, the gray-scale values of the protein bands were detected using a chemiluminescence system. Antibodies against the following proteins were used: Respiratory chain complex I/II (COX I/II) (Abcam, Cambridge, MA, USA), c-Jun *N*-terminalkinase (JNK), p-JNK, p38, p-p38, Bax, Bcl-2, cysteine containing aspartate3 (caspase3), cleaved-caspase3, β-actin (BBI Life Sciences, San Francisco, CA, USA), and Interleukin-8,10 (IL-8,10) (Wanleibio, Beijing, China).

### 4.7. Analysis of Liver Injury

Liver injury was assessed through the measurement of alanine aminotransferase (ALT), aspartate transaminase (AST) (Kinghawk Pharmaceutical, Beijing, China), superoxide dismutase (SOD), malondialdehyde (MDA), glutathione peroxidase (GSH-PX), and catalase-peroxidase (CAT) (Sigma, USA), following the manufacturer’s instructions. The detection of ALT and AST was performed using mouse sera. Ten percent of total liver tissue homogenates or cell suspension volumes were used to detect SOD, GSH-PX, CAT, and MDA.

### 4.8. Analysis of Metabolic Enzymes, Complexes 

The activity of hexokinase (HK), phosphofructokinase (PFK), lactate dehydrogenase (LDH), citrate synthase (CS), and succinate dehydrogenase (SDH) were detected in the cells and tissues, according to the manufacturer’s instructions (Sigma, USA). The activity of COX I and II were detected in cells and tissues, according to the manufacturer’s instructions (Jiancheng, Nanjing, China). 

### 4.9. Analysis of Adenosine Triphosphate (ATP) Production and Glucose Consumption

ATP production and glucose consumption were tested in cells and tissues, according to the manufacturer’s instructions (Jiancheng, China).

### 4.10. Analysis of IL-8 and IL-10 Level in Serum

The enzyme-linked immunosorbent assay (ELISA) was used to detect serum IL-8 and IL-10 in mice, according to the manufacturer’s instructions (Meimian, Shanghai, China).

### 4.11. Detection of ROS and Rhodamine 123 Accumulation

To detect ROS accumulation, we used a Reactive Oxygen Assay Kit (Beyotime, Shanghai, China) and Rhodamine 123 (Rh123) (Sigma, USA). Briefly, the cells were collected and exposed to serum-free medium containing 10 μM DCFH-DA or 5 μg/mL Rh123. After 20 min of incubation in the dark, cells were washed with DMEM three times. The fluorescence intensity of ROS or Rh 123 was measured by fluorescence microscopy and FCM with excitation wavelengths (502 and 530 nm) and emission wavelengths (488 and 505 nm). 

### 4.12. Preparation and Detection of Electron Microscopy Specimens

The digestive cells were washed twice with PBS and centrifuged to the bottom of the tube. The cell samples were double-fixed with 5% glutaraldehyde and 1% osmium acid. After being fixed, the samples were rinsed with buffer and dehydrated using a graded acetone series. The block was embedded in a porous rubber-embedded template. Following drying, slicing, and staining, the morphology and ultrastructure of the cells were observed by transmission electron microscopy (FEI, Hillsboro, OR, USA, Tecnai spirit120kv).

### 4.13. Bioinformatics

The gene expression profiles of rat liver tissues after CCl_4_ treatment were downloaded from GEO (GSE73494) [44]. We selected data for the present study in accordance with the following inclusion criteria: (1) The genes had been named publicly and (2) where the ratios of 48 h group signals to 0 h group signals were ≥2 or ≤0.5. An unsupervised hierarchical clustering analysis of genes meeting these criteria was conducted using the edgeR package (3.5.0, Berkeley Heights, NJ, USA), and several glycolysis-related genes which were expressed differentially were selected for further investigation.

### 4.14. Statistical Analysis

All experimental data was presented as the mean ± SD of independent measurements. Each experiment was performed in triplicate tests and repeated on three independent days. Data comparing two experimental conditions was statistically analyzed by *t*-test, and only results with *p* < 0.05 were considered to be statistically significant: * *p* < 0.05, ** *p* < 0.01, *** *p* < 0.001.

## 5. Conclusions

In summary, our current study demonstrated that OA can ameliorate chemical liver injury in vitro and in vivo. On one hand, OA effectively reduced the production of ROS, reduced oxidative stress injury, and protected the integrity of liver cell mitochondria. On the other hand, OA increased the production of ATP by promoting the TCA cycle and oxidative phosphorylation. Finally, OA suppressed phosphorylation of the MAPK pathway and inhibited the MAPK-mediated apoptotic pathway. Conclusively, our findings indicated a role for OA in the protection of the liver and clarified its functional mechanism.
